# Transition of Editors at the Journal of Health, Population and Nutrition

**Published:** 2007-06

**Authors:** David A. Sack

**Affiliations:** Journal of Health, Population and Nutrition and Executive Director, ICDDR,B GPO Box 128, Dhaka 1000, Bangladesh Email: riceors@yahoo.com

The Journal of Health, Population and Nutrition (JHPN), which is published by the International Centre for Diarrhoeal Disease Research, Bangladesh (ICDDR,B), evolved from the Journal of Diarrhoeal Diseases Research (JDDR) in June 2000. While the JDDR focused exclusively on diarrhoeal diseases, the JHPN broadened its view of health and illness in developing countries. This transformation coincided with my appointment as the Executive Director of the Centre and as Editor-in-Chief of the Journal, as well as with the development of a new strategic plan for the Centre. Following this June 2007 issue, the Editor-in-Chief will change again, as the new Executive Director of the Centre, Dr. Alejandro Cravioto will also become the new Editor-in-Chief for the Journal. This transition in Editors provides a time to reflect on the role the Journal has played since its evolution from the JDDR seven years ago.

The JDDR recognized that diarrhoeal diseases continue to be one of the most common causes of mortality in children. Relative to other major health problems of developing countries, such as pneumonia, malnutrition, HIV/AIDS, tuberculosis, and malaria, diarrhoeal disease ranks with a similar or greater disease burden. However, ICDDR,B recognized that diarrhoeal diseases should be understood in a larger context. The expansion of the Centre's research agenda included other important public-health challenges for developing countries, especially those issues that relate to poverty. The Centre's strategic plan identified these issues through the establishment of eight cross-cutting programmes or themes. These included child health, reproductive health, infectious diseases and vaccines, nutrition, health and family-planning systems, population sciences, poverty and health, and HIV/AIDS. Each of these themes was to be reflected in the JHPN, marking the evolution of the Centre's research priorities.

The JHPN intended to be a journal for developing-country scientists and to focus on health issues of their countries. While more prominent journals do publish articles from developing countries, the JHPN attempted to be a special venue for such manuscripts. We hoped the Journal would attract items reflecting the complex relationships between health, nutrition and population. Perhaps this goal was overly ambitious since scientific research tends to focus on a particular area, often avoiding the complexities that are so important to the overall health of populations living in developing countries. I continue to believe that this is an objective worth exploring.

Since the JHPN is targeted to developing-country scientists, we felt it important to make the Journal freely available in full text on the Internet to allow for the widest possible audience. Being indexed in PubMed, articles appearing in the Journal can easily be retrieved by anyone with access to the Internet. Through the efforts of the World Health Organization, many more journals are now available to developing-country scientists through HINARI, including JHPN. Although not all countries are able to access articles through HINARI, many more scientists and practitioners now have an opportunity to read widely as background for their own research efforts.

The JHPN is also covered by other major international indexing/abstracting systems, including Current Contents (Clinical Medicine), Elsevier Bibliographic Databases, POPLINE, Google Scholar, etc. In a recent rating by the IndexCopernicus™, the JHPN has been graded to be the 33rd among the top 100 journals covered in its database. Former BoT Chair Prof. Terry Hull, in his report on the Information Sciences Division of ICDDR,B review in 2005, lauded the Journal by calling it “The Lancet of the East.”

Since the change from the JDDR to the JHPN in June 2000, the Journal has received over 1,000 manuscripts from over 90 countries. Nearly 50% of those submitted were accepted, but with an increasing rate of submissions, the acceptance rate is decreasing. Geographically, scientists from South Asia (India, Bangladesh, Pakistan, and Nepal) submitted the largest number of manuscripts (about 40%). Scientists from Africa submitted about 20%, the largest numbers coming from Nigeria and South Africa. Others were sent from the Middle East, and Latin America as well as the North America and Europe.

Those manuscripts which were not accepted largely fell into three categories. One category included those that were potentially important nationally or locally, but not of international relevance. A second group included those that our external peer-reviewers felt did not meet the rigorous scientific standards required for an international journal. A third group of papers fell outside the scope of our journal or were on topics that we did not feel competent to review, such as the pharmacology of traditional medicines or the epidemiology of certain chronic or metabolic diseases. Clearly, chronic diseases and issues of adult health are increasingly recognized as important for developing countries; however, the JHPN has not yet addressed these in a meaningful manner. English was not the first language for many of the authors; however, we expected the authors to provide manuscripts with acceptable grammar and not requiring excessive editing.

Most (80%) of the articles were original full articles though some were scholarly reviews, letters-to-the-editor, or summaries of important meetings, especially those relevant to child health. We felt that publication of such conference summaries, such as those organized by offices of the World Health Organization, provided a rapid mechanism for disseminating the consensus of public-health experts. Some meetings, generally published months later as special reports, are not generally indexed by PubMed. By including them in the JHPN, they can be made widely accessible through subscription, many web-based indexes and HINARI.

Several issues of the Journal were devoted to special topics, such as vaccines, arsenic contamination of drinking-water, reproductive health, health equity, and healthcare use. These topics were ones that we felt deserved special attention and were also of high priorities for the Centre. The articles appearing in these special issues were peer-reviewed in the same manner as manuscripts appearing in other editions of JHPN.

We believe that the JHPN continues to serve a useful purpose of disseminating research findings of international interest, relevant to developing countries, and especially those from institutions in developing countries. It is widely known that most of the health-research funds are targeted toward diseases of the developed world while only a small portion goes for problems of developing countries, where most of the disease burden exists. This inequity is known as the 10/90 gap. A similar 10/90 gap occurs in the medical literature in which most peer-reviewed articles originate from developed-country organizations with relatively few originating from developing-country institutions where the disease burden is the greatest (G. Paraje, Sadana R, Karam G. Public health. Increasing international gaps in health-related publications. *Science* 2005; 308:959-60). Hopefully, this gap can be partially addressed through journals such as the JHPN.

I want to thank the section editors and the many reviewers who have assisted the Journal through these last seven years and to thank the many scientists who have submitted their manuscripts to the Journal. I am sure that under the new leadership of Dr. Alejandro Cravioto as Editor-in-Chief, the Journal will continue to thrive and to fulfil its mission.

